# Polr3b heterozygosity in mice induces both beneficial and deleterious effects on health during ageing with no effect on lifespan

**DOI:** 10.1111/acel.14141

**Published:** 2024-03-11

**Authors:** Gillian Borland, Stephen E. Wilkie, Jackie Thomson, Zhe Wang, Jennifer M. A. Tullet, Nazif Alic, Colin Selman

**Affiliations:** ^1^ School of Biodiversity, One Health and Veterinary Medicine University of Glasgow Glasgow UK; ^2^ Faculty of Natural Sciences University of Kent Canterbury UK; ^3^ Department of Genetics Evolution and Environment, Institute of Healthy Ageing University College London London UK; ^4^ Present address: School of Molecular Biosciences University of Glasgow Glasgow UK; ^5^ Present address: Division of Molecular Metabolism, Department of Medical Biochemistry and Biophysics Karolinska Institutet Solna Sweden

**Keywords:** Aging, C57BL/6N, healthspan, longevity, RNA polymerase III

## Abstract

The genetic pathways that modulate ageing in multicellular organisms are typically highly conserved across wide evolutionary distances. Recently RNA polymerase III (Pol III) was shown to promote ageing in yeast, *C. elegans* and *D. melanogaster*. In this study we investigated the role of Pol III in mammalian ageing using C57BL/6N mice heterozygous for Pol III (*Polr3b* ^*+/−*^). We identified sexually dimorphic, organ‐specific beneficial as well as detrimental effects of the *Polr3b* ^*+/−*^ mutation on health. Female *Polr3b* ^*+/−*^ mice displayed improved bone health during ageing, but their ability to maintain an effective gut barrier function was compromised and they were susceptible to idiopathic dermatitis (ID). In contrast, male *Polr3b* ^*+/−*^ mice were lighter than wild‐type (WT) males and had a significantly improved gut barrier function in old age. Several metabolic parameters were affected by both age and sex, but no genotype differences were detected. Neither male nor female *Polr3b* ^*+/−*^ mice were long‐lived compared to WT controls. Overall, we find no evidence that a reduced Pol III activity extends mouse lifespan but we do find some potential organ‐ and sex‐specific benefits for old‐age health.

## Graphical abstract

RNA polymerase III (Pol III) promotes ageing in yeast, *C*. *elegans* and *D*. *melanogaster*. We investigated the role of Pol III in mammalian ageing using male and female C57BL/6N mice heterozygous for the Pol III catalytic subunit Polr3b (Polr3b^+/−^). Polr3b^+/−^ mice were not long‐lived but we did observe some organ‐ and sex‐specific benefits on age‐related health.

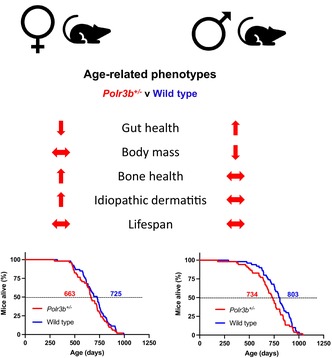

The ageing process is associated with a profound decline in physiological function and increased prevalence in multiple pathologies (Figueira et al., 2016). It is well established that lifespan can be extended through dietary, pharmacological, and genetic means (Fontana & Partridge, 2015; Gems & Partridge, 2013; Mannick & Lamming, 2023), with several of these interventions also delaying and/or reducing age‐related pathology (Selman & Withers, 2011). RNA polymerase III (Pol III) is one of three nuclear RNA polymerases found in eukaryotes. It transcribes a number of short non‐coding RNAs (e.g., tRNAs, snRNAs, 5S rRNA (Kulaberoglu et al., 2021)), and is estimated to account for ~15% of total cellular transcription (Moir & Willis, 2013). Pol III inhibition extends lifespan in yeast, *C. elegans* and *D. melanogaster*, acting through the intestine/intestinal stem cells to achieve this in worms and flies respectively (Filer et al., 2017). Inhibition also preserves age‐related health in flies and acts downstream of mTORC1 (Filer et al., 2017).

In mammals, Pol III consists of 17 subunits, of which Polr3a and Polr3b are the largest and form the catalytic subunit of the polymerase (Choquet et al., 2019; Kulaberoglu et al., 2021). Given that Pol III can modulate lifespan in invertebrate models and that this phenotype appears not to be subunit‐specific in flies (Filer et al., 2017), we examined longevity and aspects of age‐related health in mice heterozygous for *Polr3b* (*Polr3b* ^*+/−*^) which encodes the second largest catalytic subunit of Pol III (Figure S1A,B); global homozygous loss of *Polr3b* causes embryonic lethality (see Methods Data S1). Mice bred with expected Mendelian frequencies (Figure 1a) and hepatic expression of *Polr3b* was reduced in both female and male *Polr3b* ^*+/−*^ mice (Figure 1b; Hepatic POLR3b protein levels were also reduced in female, but not male, mice) (Figure S2A,B). Performing extensive phenotyping at different ages, we observed both beneficial and detrimental, sexually dimorphic, organ‐specific effects of the heterozygous *Polr3b* mutation on health. Akin to humans, mice exhibit age‐related bone loss (Jilka, 2013) and this loss can be assessed by microCT (Selman et al., 2009). Female *Polr3b* ^*+/−*^ mice showed increased trabecular bone relative to WT females particularly evident at mid‐life (Figure 1c–e; Figure S3), similar to our previously findings in long‐lived IIS and mTOR mutants (Selman et al., 2008, 2009), indicating that reduced Pol III activity may help maintain bone health during ageing in female mice. No phenotypic differences in bone characteristics were observed in males (Figure S4A–C).

**FIGURE 1:**
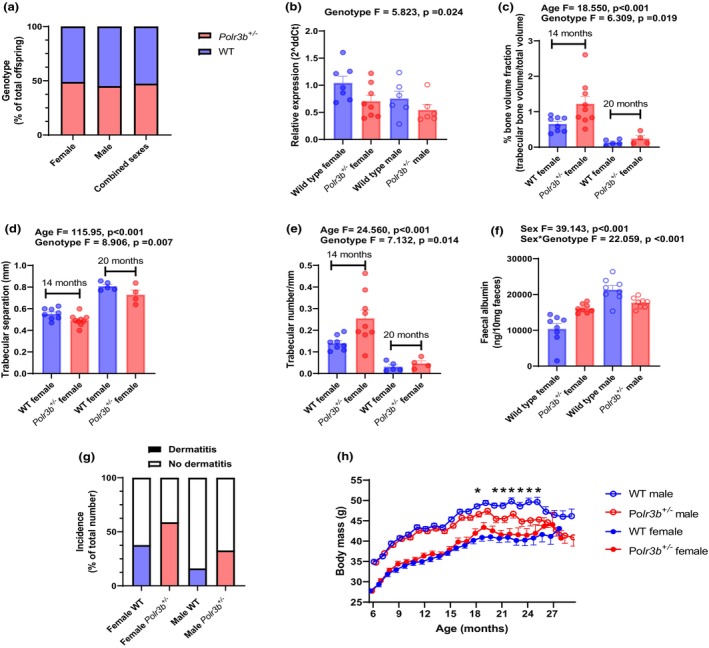
(a) Mendelian frequencies of mice born from heterozygous *Polr3b* ^*+/−*^ x wild‐type (WT) parents. (b) Hepatic *Polr3b* expression in 14‐month‐old mice. (c) Percentage bone volume, (d) Trabecular separation, (e) Trabecular number in female mouse femurs at 14 and 20 months. (f) Faecal albumin levels in female and male mice at 22 months. (g) Percentage of mice (of total number) presenting with idiopathic dermatitis. (h) Body mass (mean ± SEM) across the life‐course in female and male WT and *Polr3b* ^*+/−*^ mice. Histograms denote mean ± SEM, with sample sizes indicated by individual points within a group. For (b–f), a two‐way ANOVA was used to test for age/sex and genotype effects (both main and interaction effects). Only significant main and/or interaction effects are reported within the figures.

With age, the barrier function of the mouse gut becomes compromised, and this leakiness can be observed through an increase in faecal albumin (Wang et al., 2021). In contrast to the preserved gut barrier function observed in older flies with attenuated Pol III activity (Filer et al., 2017), female *Polr3b* ^*+/−*^ mice showed increased gut permeability compared to age‐matched WT controls (Figure 1f). These females were also susceptible to ID (Figure 1g). On the other hand, *Polr3b* ^*+/−*^ males were lighter (Figure 1h) and their gut barrier function was preserved in old age (Figure 1f), relative to WT controls. For several other metabolic phenotypes (Figure S4d–i) and for grip strength (Figure S4j), no genotypic differences were detected at any age in either sex, although several significant age and sex effects were seen.

We evaluated lifespan in female and male C57BL/6N WT mice compared to *Polr3b* ^*+/−*^ mice. Combined data from both sexes (Figure 2a) showed no significant effect of genotype on median or maximum lifespan (oldest 10% of cohort, Figure S5A; Table S1). Similarly, when each sex was analysed separately neither female nor male *Polr3b* ^*+/−*^ mice were long‐lived (Figure 2b,c; Figure S5B,C: Table S1). Male WT mice lived significantly longer than female WT mice (*X* ^2^ = 10.420, *p* = 0.001), with a similar sex‐specific trend seen in *Polr3b* ^*+/−*^ mice (*X* ^2^ = 3.810, *p* = 0.051). Median lifespan of our C57BL/6N WT mice was shorter than our previously published data for C57BL/6J mice (Selman et al., 2008, 2009). C57BL/6J and 6N mice differ in a range of metabolic parameters (Selman & Swindell, 2018), but there is a current dearth of published lifespan data for C57BL/6N mice. However, our lifespans compare favourably with published lifespans for this strain (Reid et al., 2023; Tang et al., 2021). No genotype difference in cancer incidence upon post‐mortem was identified in our ageing cohorts (Figure 2d). Note that censoring female mice euthanised for ID did not alter the lifespan outcome (Figure S5D; *X* ^2^ = 0.001, *p* = 0.970).

**FIGURE 2:**
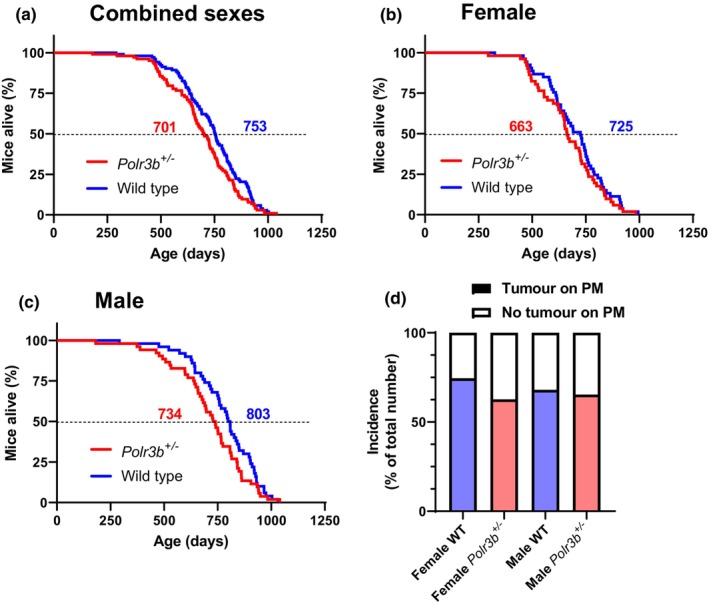
(a) Kaplan–Meier survival curves for combined female and male wild‐type (WT) and *Polr3b* ^*+/−*^ mice (log‐rank *X* ^2^ = 3.084, *p* = 0.079, *n* = 103 for WT, *n* = 103 for *Polr3b* ^*+/−*^). Survival curves for female (b; log‐rank *X* ^2^ = 1.040, *p* = 0.308, *n* = 53 for WT, *n* = 51 for *Polr3b* ^*+/−*^) and male (c; log‐rank *X* ^2^ = 2.355, *p* = 0.125, *n* = 50 for WT, *n* = 52 for *Polr3b* ^*+/−*^) mice. Numbers denote median lifespan (days). (d) Percentage of mice presenting post‐mortem with macroscopic tumours.

Overall, we observed an indication of organ‐ and sex‐specific benefits for old‐age health achieved by a partial loss‐of‐function in Pol III with no effect on lifespan. In support, pathogenic conditions associated with Pol III appear to show pronounced tissue‐specific responses following perturbation in Pol III transcription (Watt et al., 2023). The difference to the net beneficial effect on invertebrate lifespan may result from a more complex physiological role of Pol III in mammals. For example, Pol III plays a critical noncanonical role in viral and bacterial DNA sensing within the innate immune response (Chiu et al., 2009). It is possible that the appropriate response to bacterial and viral challenge at the skin surface is impaired in female *Polr3b* ^*+/−*^ mice causing ID. Our mice were heterozygous for *Polr3b*, and at the level of both protein and gene expression the reduction in *Polr3b* observed was variable both within and between sexes. It is possible that a more substantial reduction in Polr3b expression in specific cell types is required to obtain a longevity phenotype. Still, the observation of some beneficial effects, for example, on bone health in females, suggests that cell‐type specific attenuation of Pol III function may have positive effect on aspects of mammalian ageing, as indicated by a recent analysis of human genetic data (Javidnia et al., 2022).

## AUTHOR CONTRIBUTIONS

Nazif Alic, Jennifer M.A. Tullet, and Colin Selman conceived the study and obtained the funding. Gillian Borland, Stephen E. Wilkie, Jackie Thomson, Zhe Wang, and Colin Selman performed the experiments. Gillian Borland and Colin Selman analysed the data. Gillian Borland, Nazif Alic, Jennifer M.A. Tullet, and Colin Selman wrote the manuscript with contributions from all authors.

## FUNDING INFORMATION

This work was funded by the Biotechnology and Biological Sciences Research Council (BBSRC) grant BB/S014357/1 to CS, JMAT and NA. SEW was funded through a Medical Research Council Doctoral Training Program to CS (Reference MR/N013166/1).

## CONFLICT OF INTEREST STATEMENT

The authors declare no conflicts of interest.

## Supporting information


Data S1.

Figure S1.

Table S1.
Table S2.

Table S3.

## Data Availability

The data that support the findings of this study are available from the corresponding author upon reasonable request. All individual lifespan data are included within the supplementary material Table S3).
