# Supplementary material for: Polr3b heterozygosity in mice induces both beneficial and deleterious effects on health during ageing with no effect on lifespan
Source: Aging Cell. 2024 Mar 11;23(5):e14141. doi: 10.1111/acel.14141 (PMC11113255; doi:10.1111/acel.14141)
Supplement: Supplementary file 1 — Data S1. [file ACEL-23-e14141-s002.docx]

**MATERIALS AND METHODS**

**Mouse model and husbandry**

The *C57BL/6NCrl-Polr3b^em7(IMPC)Tcp/Tcp^* mouse line was generated (Fig. S1A shows CRISPR/Cas9 targeting strategy) as part of the NorCOMM2 project funded by Genome Canada and the Ontario Genomics Institute (OGI-051) at the Toronto Centre for Phenogenomics (<http://phenogenomics.ca>). It was obtained from the Canadian Mouse Mutant Repository as part of the Knockout Mouse Program (KOMP2) (Lloyd, 2011). Heterozygous mice (hereafter *Polr3b*^+/-^) were bred in-house with non-transgenic C57Bl/6N wild type (WT) mice to maintain the line on a heterozygous background. It has previously been reported by the International Mouse Phenotyping Consortium (Toronto Centre for Phenogenomics) through a viability primary screen that homozygosity for the KO lead to preweaning lethality in both male and female mice with complete penetrance prior to organogenesis and prior to tooth bud stage. <https://www.mousephenotype.org/data/charts?accession=MGI:1917678&parameter_stable_id=IMPC_VIA_001_001&parameter_stable_id=IMPC_VIA_063_001&parameter_stable_id=IMPC_VIA_064_001&parameter_stable_id=IMPC_VIA_065_001&parameter_stable_id=IMPC_VIA_066_001&parameter_stable_id=IMPC_VIA_067_001>

Experimental *Polr3b^+/-^* offspring were generated by mating *Polr3b^+/-^* mice with C57BL/6N mice; to avoid any potential carry-over effect in experimental mice, WT offspring were generated by mating WT mice generated from *Polr3b^+/-^* x C57BL/6N matings with C57BL/6N mice. Mice were genotyped from ear biopsies either in-house by PCR (Fig. S1A and S1B), or by qPCR using an external provider (Transnetyx Inc, Cordova, TN, USA). In-house PCR was carried out on genomic DNA using forward primers that recognised both the WT and knockout alleles, while reverse primers were sited over the location of the 8bp insertion in the knockout allele and were therefore specific for the WT or knockout allele (Fig. S1A and 1B). PCR was set up using 12.5µl DreamTaq Green (ThermoFisher, Inchinnan, UK), 2.5µl each primer, 0.75µl genomic DNA in a total of 25µl. Amplification was carried out in a MiniAmp Plus thermal cycler (ThermoFisher, Inchinnan, UK) using the following conditions: 95˚C for 10 minutes; 35 cycles of 95˚C for 30 seconds, 53˚C for 35 seconds and 72˚C for 65 seconds; 72˚C for 5 minutes. PCR products were run on a 2% agarose gel and visualised using a BioRad Gel Doc XR+ (BioRad, Watford, UK). Primer sequences are in Table S2. Mendelian frequencies were calculated using data from breeding cages used to generate the longevity cohort. Six breeding cages contained two C57BL/6N females and one *Polr3b^+/-^* male; an additional six cages contained two *Polr3b^+/-^* females and one C57BlL6N male. The first litters from each of these 12 cages contained a total of 148 pups (60 female and 88 male) that survived until weaning, and genotyping results for these pups were used to calculate Mendelian frequencies.

Mice were group housed under barrier conditions (typically 2-5 same-sex littermates per cage from weaning) in individually ventilated cages at approximately 22˚C on a 12L/12D cycle (lights on 7.00am – 7.00pm) with *ad libitum* access to water and standard mouse chow (CRM(P), Research Diets Services, LBS Biotech, UK; Atwater Fuel Energy-protein 22%, carbohydrate 69%, fat 9%). Mice were health-checked daily and weighed monthly (weekly for mice after reaching 15 months on age) using an Ohaus CX221 portable balance; mice showing signs of pathology or acute weight loss were subject to enhanced monitoring and assessed using a scoring system to measure cumulative harm from normal age-related changes and pathological changes. Mice were scored on general physical appearance (e.g. presence of tumours/dermatitis, piloerection), breathing, activity, changes in body mass and changes in body condition. Mice identified with ID were given standard treatment: lesions were treated with green clay (montmorillonite) daily and nails were trimmed. Mice were weighed and ID was monitored daily until they either recovered or met a humane end point. Mice were euthanised if they reached any of the following humane end-points: mouse reached a threshold score; mouse with severe dermatitis not responding to treatment; mouse unable to weight-bear on hind limbs, affecting its ability to feed/drink normally; mouse with a tumour that caused rapid reduction in body mass or body condition, or that was ulcerated, or that interfered with normal movement or behaviour; mouse with gasping or rapid breathing; mouse with recent weight loss >20%, or >10% and not responsive to treatment; mouse with a body condition score of 1. Necropsies were carried out where possible on mice that were euthanised or died spontaneously. In the mice used in the longevity group, 94% of the total number of mice reached our humane end points and were euthanised, while 6% died spontaneously, with no differences observed between genotypes on whether mice were euthanised or not. All experimental procedures were approved by The University of Glasgow Animal Welfare and Ethical Review Board, under a UK Home Office Project Licence (PDBDC7568) and following the “principles of laboratory animal care” (NIH Publication No. 86–23, revised 1985).

**Longevity study**

Mice included in the longevity study were held in the same room throughout the experiment. These mice were subject to non-invasive healthspan measurements (frailty index, grip strength) in addition to regular body mass measurements throughout life but were otherwise left undisturbed until they reached our humane end points or died spontaneously. Where a mouse was euthanised after reaching a humane end point, the date of cull was used as the date of death. Where a single surviving female mouse was left in a cage it was transferred to a cage of female mice of the same genotype within the longevity study. Survival was assessed in 104 female mice (n= 53 for WT, n= 51 for *Polr3b^+/-^*) and 102 male mice (n= 50 for WT, n= 52 for *Polr3b^+/-^*). See Table S3 for the raw lifespan data for all mice in this study (where F= female and M = male). Where ID deaths were censored from survival curves, only those mice euthanised for severe dermatitis (or severe dermatitis combined with weight loss with no other identifiable cause) meeting a humane end point with no additional illnesses were censored.

**Cross-sectional studies**

Additional cohorts of male and female *Polr3b^+/-^* and C57BL/6N WT mice were generated for analysis of young (3-4 months), middle-aged (12-14 months) and old (20 months) mice. These mice were not included in the longevity analysis and were subject to glucose tolerance tests and fed blood glucose measurements in the month before they were culled at the appropriate age for harvesting of tissue samples and plasma.

**Food intake**

Food intake under *ad libitum* conditions was measured in male and female WT and *Polr3b^+/-^* mice at 12 months of age and collected across a 1-week period. Food intake was calculated as grams of food consumed per day expressed per gram of body mass.

**Fed/fasted blood glucose and glucose tolerance measurements**

Fed blood glucose measurements were taken between 9am and 11am by venesection of caudal vein and analysing a drop of blood with a OneTouch Ultra Easy glucometer (Lifescan, CA, USA). Glucose tolerance tests (GTT) were performed as described previously (Crowe et al., 2020). Mice were fasted from approximately 1830 to 0830, weighed, warmed in a heated cabinet at 38˚C and then a baseline fasted blood glucose concentration was recorded as described above for fed blood glucose. Mice were injected intraperitoneally with 2g glucose per kg body mass using a sterile-filtered solution of 20% glucose in water. Blood glucose levels were measured at 15-, 30-, 60- and 120-minutes post-glucose injection by gently reopening the caudal vein cut and taking sequential blood samples. The area under the curve (AUC) was calculated using GraphPad Prism (version 9; GraphPad, San Diego, CA, USA).

**Fasted blood insulin and IGF-1 levels**

For plasma insulin (Merck Millipore, Watford, UK) and plasma IGF-1 (R&D Systems, Abingdon, UK), mice were culled by cervical dislocation after overnight fast and blood was immediately collected and plasma prepared by centrifugation at 2500g for 7 minutes at room temperature followed by collection of the supernatant and storage at -80˚C. Samples were assayed in mouse insulin (Merck Millipore, Watford, UK) and mouse IGF-1 (R&D Systems, Abingdon, UK) ELISAs according to the manufacturer’s instructions.

**Faecal albumin levels**

Fresh faecal pellets were collected onto ice from individual unfasted mice placed in a sterile, empty cage and were then stored at -80˚C until analysis. Faecal pellets were weighed and homogenised in assay dilution buffer from mouse albumin ELISA (Bethyl Laboratories Mouse Albumin ELISA kit, Cambridge Biosciences, UK) and diluted to 100µl per mg faecal weight then centrifuged at 1200g for 5 minutes at 4˚C. The supernatant was assayed at a 1:40 dilution in the ELISA according to manufacturer’s instructions.

**Grip strength**

Grip strength was measured using a previously described protocol (Bellantuono et al., 2020). Briefly, individual mice were removed from the home cage and allowed to grip a triangular bar attached to the grip strength meter (Ugo Basile, Gemonio, Italy) with front paws only; mice were pulled away from the bar until they lost grip and maximum force applied to the bar was recorded. The test was repeated 3-5 times per mouse at >1-minute intervals and the highest value recorded for each mouse was used for analysis.

**MicroCT**

Individual femurs were scanned using a Skyscan 1072 desktop CT scanner (Skyscan Bruker, Kontich, Belgium) at 50 kV, 189 mA using a 0.5mm aluminium filter (Harnett et al., 2022). The specimens were rotated 180˚ with a rotation step of 0.68˚ and an exposure time of 6.3 s. The overall scanning time was approximately 40 min per specimen. All specimens were scanned with a pixel size of 5 mm. The scans were reconstructed using NRecon 1.4.4 software (Skyscan Bruker). The slices were converted into an 8-bit BMP output format and the values within the dynamic range were mapped into grey levels [0–255].

**RT-qPCR**

Liver samples from 12 month old male and female *Polr3b^+/-^* and WT mice were snap-frozen immediately following schedule 1 killing and stored at -70˚C. RNA was prepared using the RNeasy Mini kit (Qiagen, Manchester, UK) and cDNA was prepared using Invitrogen M-MLV Reverse Transcriptase (ThermoFisher, Inchinnan, UK), both according to manufacturer’s instructions; resulting cDNA was diluted in PCR-grade H_2_O (Qiagen, Manchester, UK) to 12.5ng/μl. Quantitative PCR was carried out on 1μl cDNA, 10μl Applied Biosystems Fast Sybr Green Master Mix (ThermoFisher, Inchinnan, UK), 0.5μl of a 10μM stock of each primer and 8μl of PCR-grade H_2_O per well. Amplification using primer sets for B2M, HMBS, YWHAZ and Polr3b was carried out on a 7500 Fast Real Time PCR system (ThermoFisher, Inchinnan, UK) using the following cycling conditions: 95˚C for 10 min; 40 cycles of 95˚C for 15 seconds, 60˚C for 60 sec, 72˚C for 60 sec; and followed by a standard melt curve. Expression was calculated by the ΔΔCt method and results expressed as fold change (2^-ΔΔCt^). Primer sequences are shown in Table S2.

**Western blotting**

Liver samples from 5 month old male and female *Polr3b^+/-^* and WT mice were snap-frozen immediately following schedule 1 killing and stored at -70˚C. Samples were subsequently homogenised in RIPA buffer (50mM Tris pH8.0, 150mM NaCl, 1% Triton X-100, 0.5% sodium deoxycholate, 0.1% SDS) containing protease inhibitors (Roche Complete Mini protease inhibitor cocktail; Merck, Gillingham, UK) and phosphatase inhibitors (Roche PhosSTOP, Merck, Gillingham, UK) using a pre-chilled glass homogeniser. Homogenised samples were centrifuged at 13000rpm for 20 minutes in a pre-chilled microfuge and the supernatant was collected, assayed for total protein content by Pierce BCA assay (Thermo Fisher, Inchinnan, UK) and stored at -20˚C. Western blots were performed largely as previously described (Wilkie et al., 2023; Wilkie et al., 2020). Briefly, an equal amount of protein from each sample was separated on a Mini-protean TGX 4-20% gel (Bio-Rad, Watford, UK) and transferred to nitrocellulose. The blot was stained for total protein as a loading control using Revert 700 Total Protein Stain (Li-Cor, Cambridge, UK) then blocked in 5% dried skimmed milk powder in TBS-T. The blot was exposed to primary (anti-Polr3b Cat No. PA5-39141, Thermo Fisher, Inchinnan, UK) and secondary (anti-rabbit IgG-Alexa680 Cat No. ab186696; Abcam, Cambridge, UK) antibodies before imaging on a Li-cor Odyssey® CLx imaging system (Li-Cor, Cambridge, UK) and quantitation using ImageStudioLite (Li-Cor, Cambridge, UK).

**Statistical analysis**

All statistical analyses described were performed using IBM Corp. SPSS Statistics for Windows (version 26; Armonk, NY, USA) and GraphPad Prism (version 9; GraphPad, San Diego, CA, USA) software, using unpaired *t* test, one-way analysis of variance, or 2-way ANOVA where appropriate. Our 2-way ANOVA approach examined genotype (wild type, *Polr3b^+/-^*), sex (female, male), and in some instances age (14 months, 20 months) as main (fixed) factors, also testing interaction effects. In all cases, where non-significant interaction effects (*p* > 0.05) were identified, these were subsequently removed, and the analysis rerun with main effects to obtain the best-fitted model. In all cases, only main effects or interaction effects where p<0.05 were reported. The ROUT test was used to identify outliers (alpha = 0.05) and all data was tested for normality using the Kolmogorov-Smirnov test. Kaplan-Meier survival curves were generated from the known birth and death dates of each individual mouse as previously described (Crowe et al., 2020; Selman et al., 2009), with the log-rank test was used to evaluate statistical differences between experimental groups. Minimum/maximum life span was calculated as the mean age of the youngest/oldest 10% of mice per genotype. Results are reported as means ± se except where indicated. *P* < 0.05 was regarded as statistically significant.

**References**

Bellantuono, I., de Cabo, R., Ehninger, D., Di Germanio, C., Lawrie, A., Miller, J., . . . Lamming, D. W. (2020). A toolbox for the longitudinal assessment of healthspan in aging mice. *Nat Protoc, 15*(2), 540-574. doi:10.1038/s41596-019-0256-1

Crowe, J., Lumb, F. E., Doonan, J., Broussard, M., Tarafdar, A., Pineda, M. A., . . . Harnett, M. M. (2020). The parasitic worm product ES-62 promotes health- and life-span in a high calorie diet-accelerated mouse model of ageing. *PLoS Pathog, 16*(3), e1008391. doi:10.1371/journal.ppat.1008391

Harnett, M. M., Doonan, J., Lumb, F. E., Crowe, J., Damink, R. O., Buitrago, G., . . . Harnett, W. (2022). The parasitic worm product ES-62 protects the osteoimmunology axis in a mouse model of obesity-accelerated ageing. *Front Immunol, 13*, 953053. doi:10.3389/fimmu.2022.953053

Lloyd, K. C. (2011). A knockout mouse resource for the biomedical research community. *Ann N Y Acad Sci, 1245*, 24-26. doi:10.1111/j.1749-6632.2011.06311.x

Selman, C., Tullet, J. M., Wieser, D., Irvine, E., Lingard, S. J., Choudhury, A. I., . . . Withers, D. J. (2009). Ribosomal protein S6 kinase 1 signaling regulates mammalian life span. *Science, 326*(5949), 140-144. doi:10.1126/science.1177221

Wilkie, S. E., Marcu, D. E., Carter, R. N., Morton, N. M., Gonzalo, S., & Selman, C. (2023). Hepatic hydrogen sulfide levels are reduced in mouse model of Hutchinson-Gilford progeria syndrome. *Aging (Albany NY), 15*(12), 5266-5278. doi:10.18632/aging.204835

Wilkie, S. E., Mulvey, L., Sands, W. A., Marcu, D. E., Carter, R. N., Morton, N. M., . . . Selman, C. (2020). Strain-specificity in the hydrogen sulphide signalling network following dietary restriction in recombinant inbred mice. *Geroscience, 42*(2), 801-812. doi:10.1007/s11357-020-00168-2
