# Supplementary material for: Polr3b heterozygosity in mice induces both beneficial and deleterious effects on health during ageing with no effect on lifespan
Source: Aging Cell. 2024 Mar 11;23(5):e14141. doi: 10.1111/acel.14141 (PMC11113255; doi:10.1111/acel.14141)
Supplement: Supplementary file 2 — Figure S1. [file ACEL-23-e14141-s004.pptx]

## Slide 1
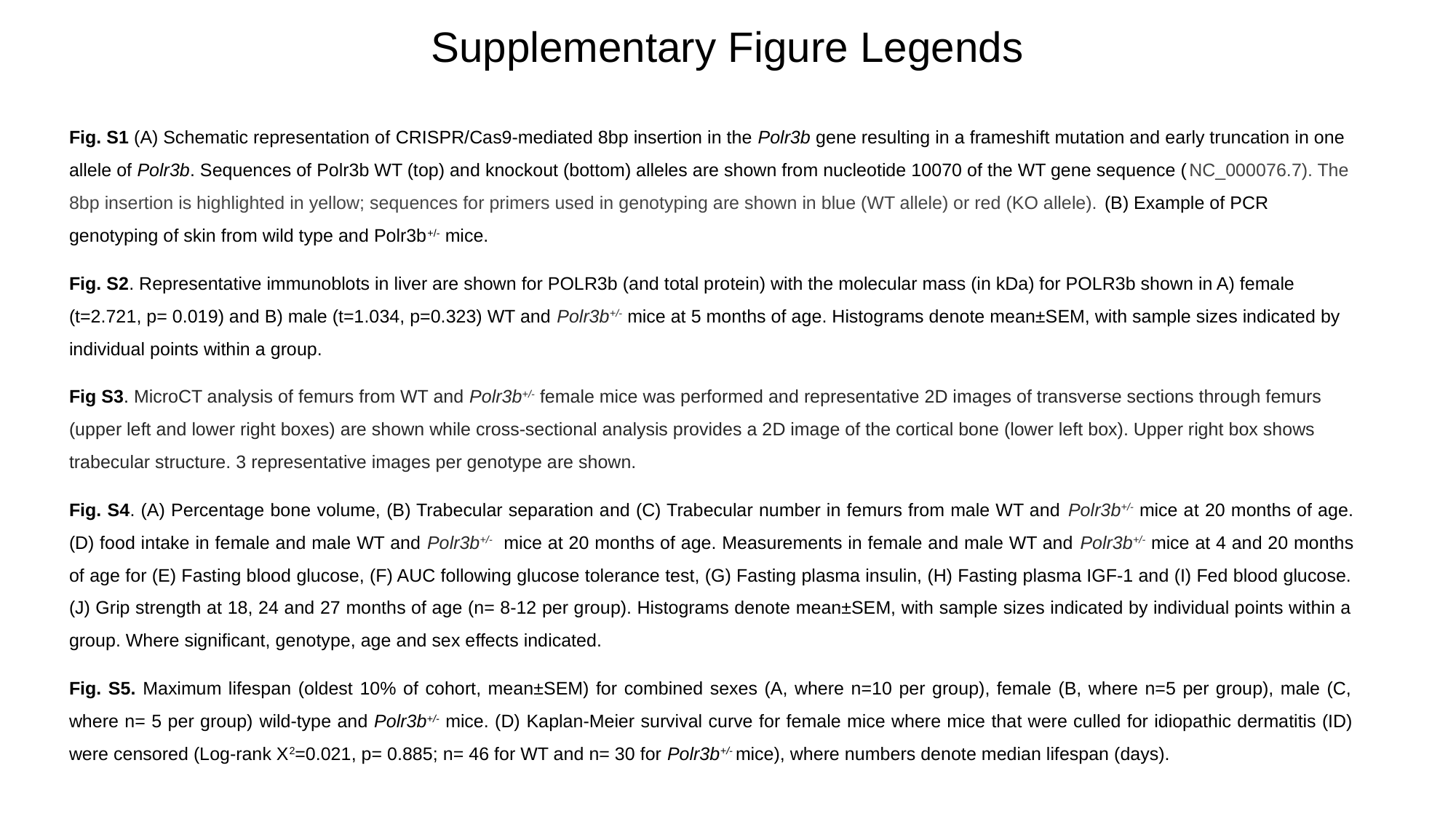

# Supplementary Figure Legends
Fig. S1 (A) Schematic representation of CRISPR/Cas9-mediated 8bp insertion in the Polr3b gene resulting in a frameshift mutation and early truncation in one allele of Polr3b. Sequences of Polr3b WT (top) and knockout (bottom) alleles are shown from nucleotide 10070 of the WT gene sequence (NC_000076.7). The 8bp insertion is highlighted in yellow; sequences for primers used in genotyping are shown in blue (WT allele) or red (KO allele). (B) Example of PCR genotyping of skin from wild type and Polr3b+/- mice.
Fig. S2. Representative immunoblots in liver are shown for POLR3b (and total protein) with the molecular mass (in kDa) for POLR3b shown in A) female (t=2.721, p= 0.019) and B) male (t=1.034, p=0.323) WT and Polr3b+/- mice at 5 months of age. Histograms denote mean±SEM, with sample sizes indicated by individual points within a group.
Fig S3. MicroCT analysis of femurs from WT and Polr3b+/- female mice was performed and representative 2D images of transverse sections through femurs (upper left and lower right boxes) are shown while cross-sectional analysis provides a 2D image of the cortical bone (lower left box). Upper right box shows trabecular structure. 3 representative images per genotype are shown.
Fig. S4. (A) Percentage bone volume, (B) Trabecular separation and (C) Trabecular number in femurs from male WT and Polr3b+/- mice at 20 months of age. (D) food intake in female and male WT and Polr3b+/- mice at 20 months of age. Measurements in female and male WT and Polr3b+/- mice at 4 and 20 months of age for (E) Fasting blood glucose, (F) AUC following glucose tolerance test, (G) Fasting plasma insulin, (H) Fasting plasma IGF-1 and (I) Fed blood glucose. (J) Grip strength at 18, 24 and 27 months of age (n= 8-12 per group). Histograms denote mean±SEM, with sample sizes indicated by individual points within a group. Where significant, genotype, age and sex effects indicated.
Fig. S5. Maximum lifespan (oldest 10% of cohort, mean±SEM) for combined sexes (A, where n=10 per group), female (B, where n=5 per group), male (C, where n= 5 per group) wild-type and Polr3b+/- mice. (D) Kaplan-Meier survival curve for female mice where mice that were culled for idiopathic dermatitis (ID) were censored (Log-rank X2=0.021, p= 0.885; n= 46 for WT and n= 30 for Polr3b+/- mice), where numbers denote median lifespan (days).

## Slide 2
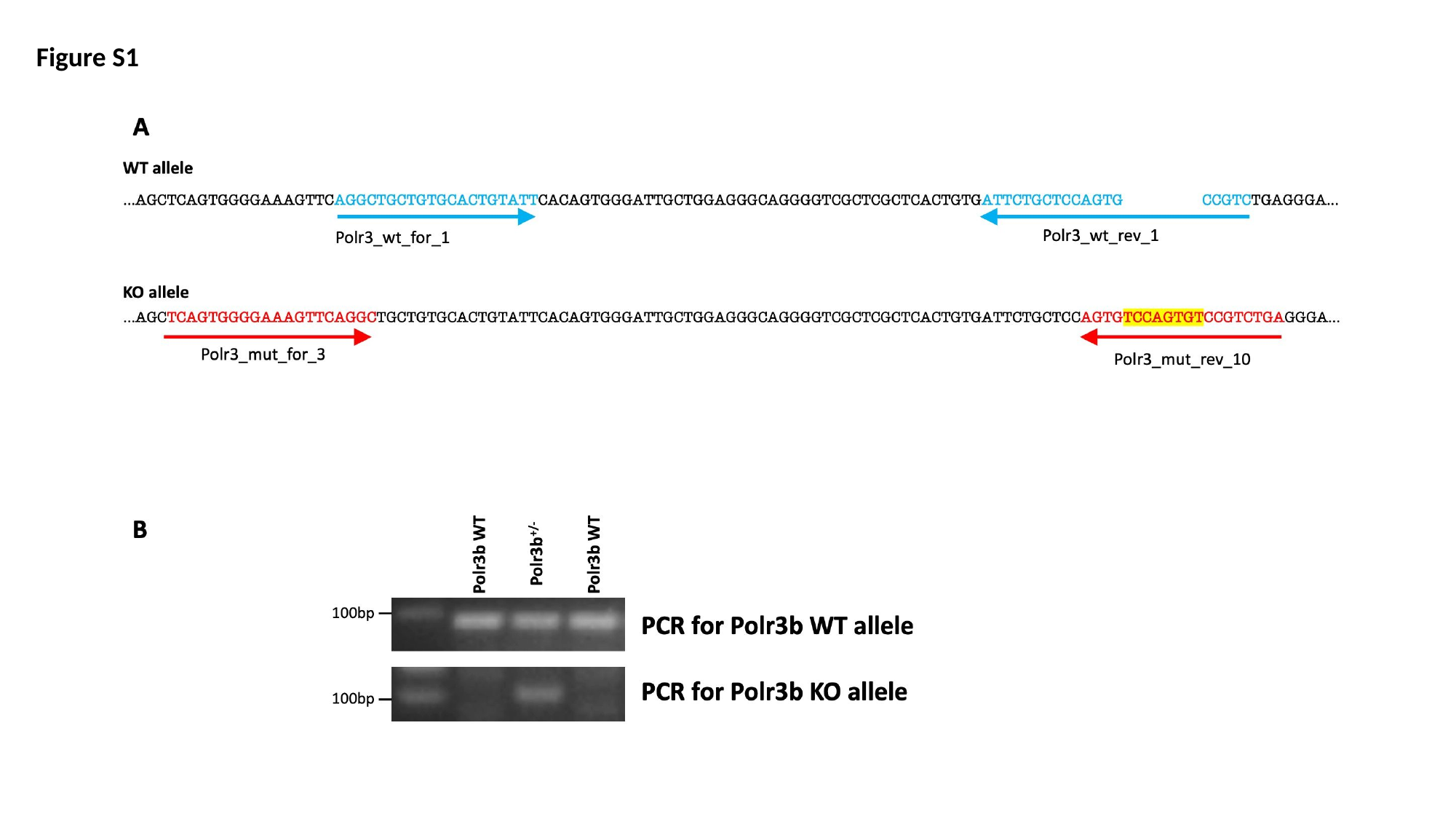

Figure S1

## Slide 3
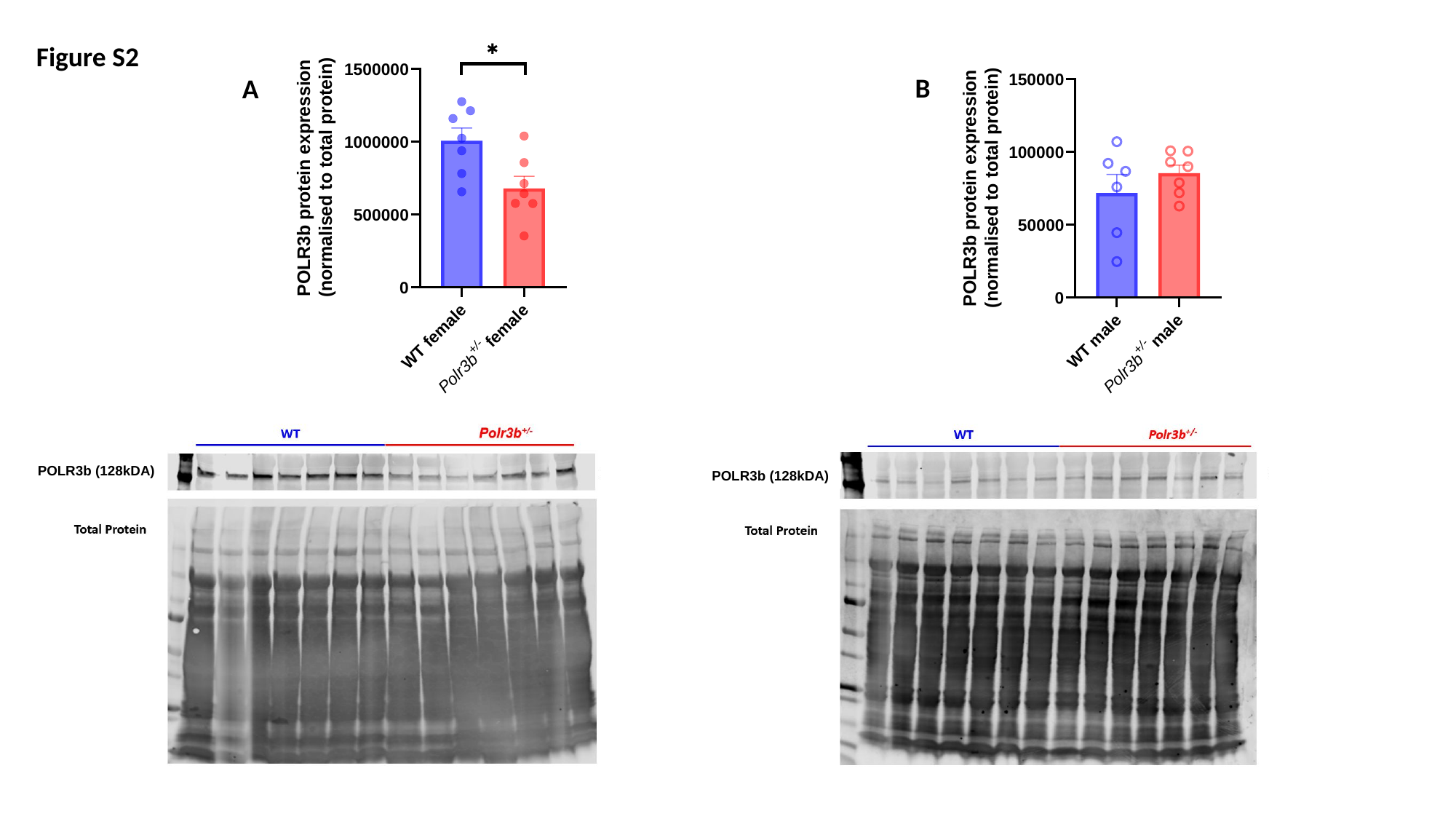

Figure S2
B
A
POLR3b (128kDA)
POLR3b (128kDA)

## Slide 4
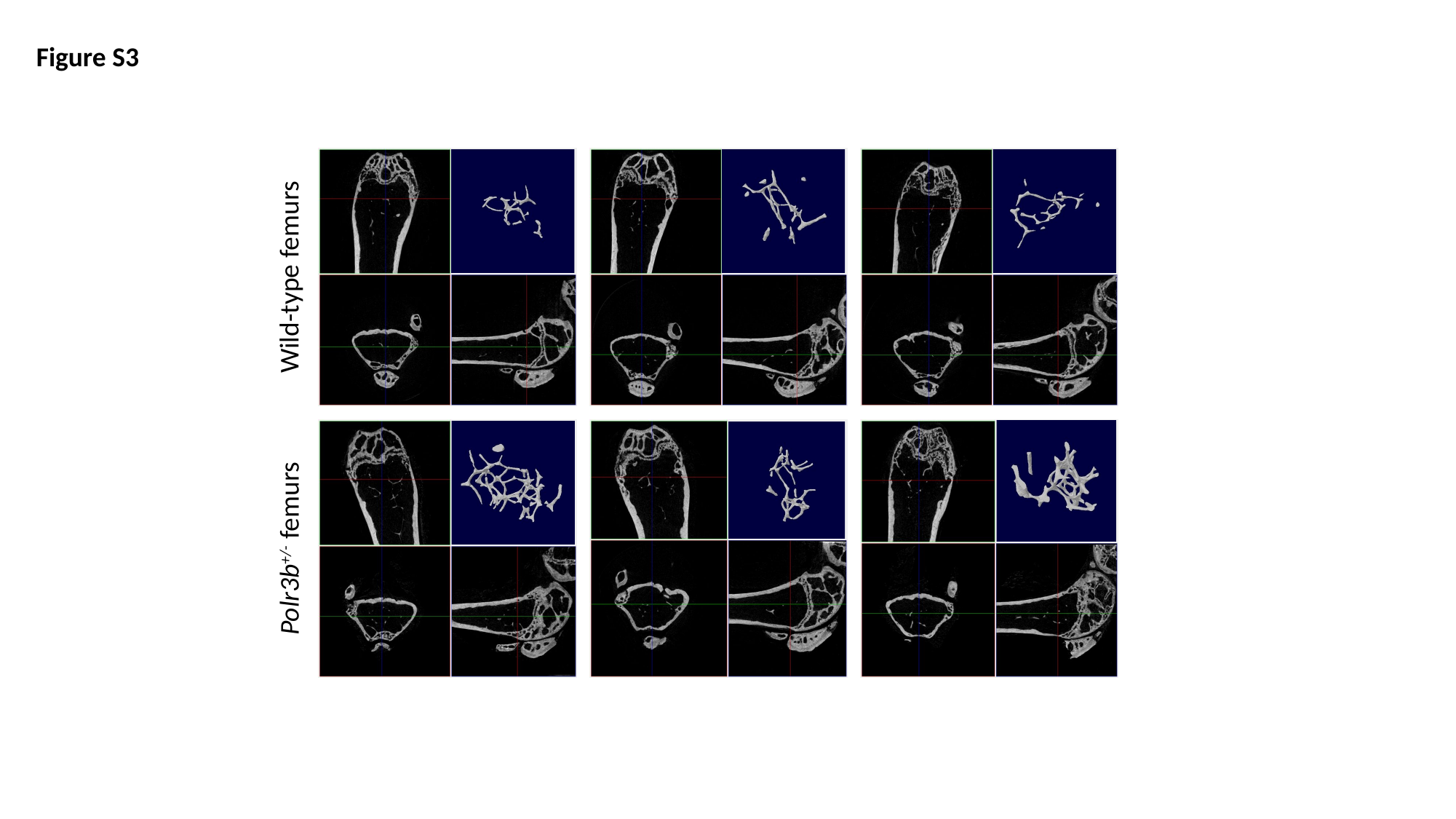

Figure S3
Wild-type femurs
Polr3b+/- femurs

## Slide 5
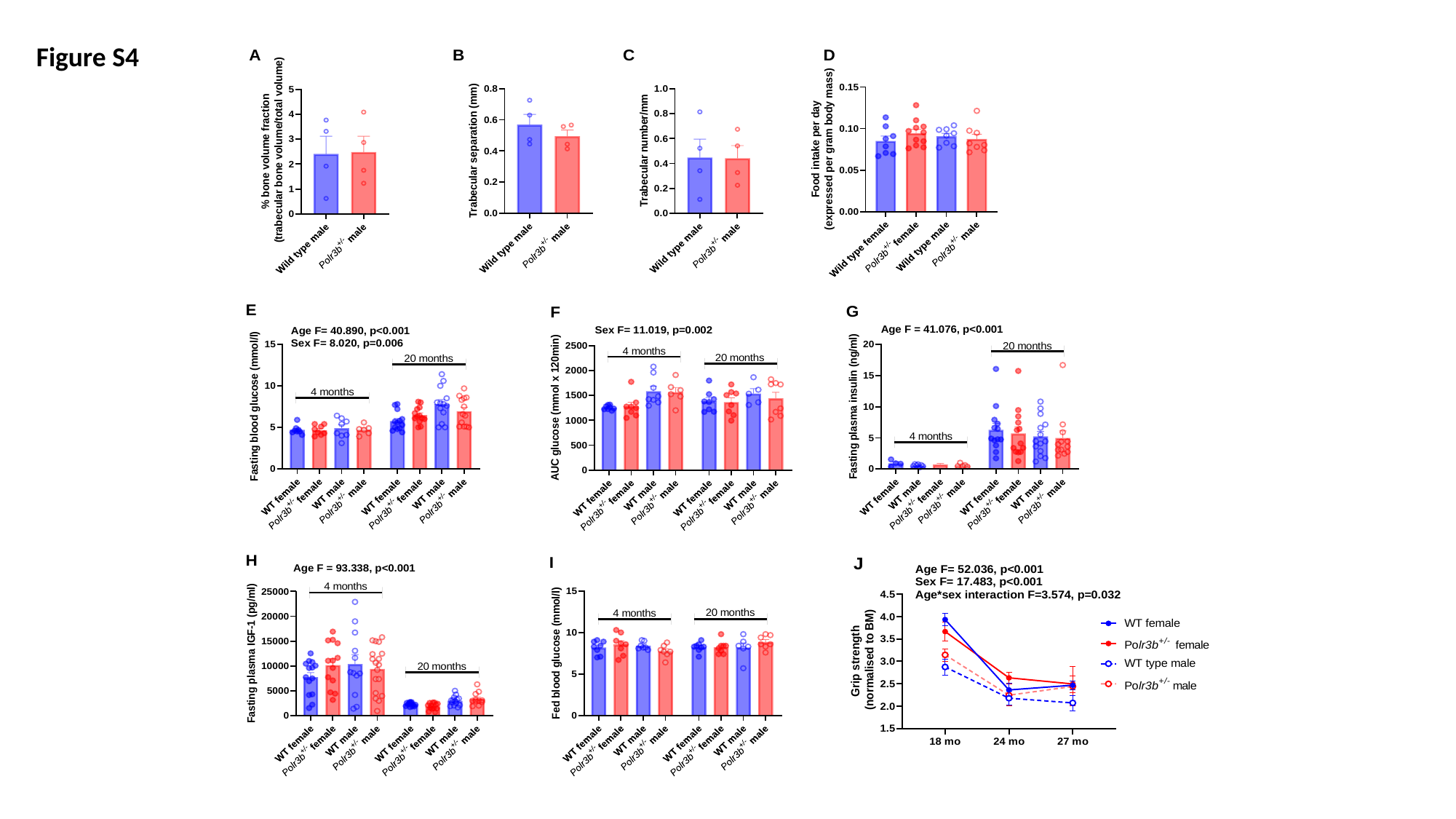

Figure S4

## Slide 6
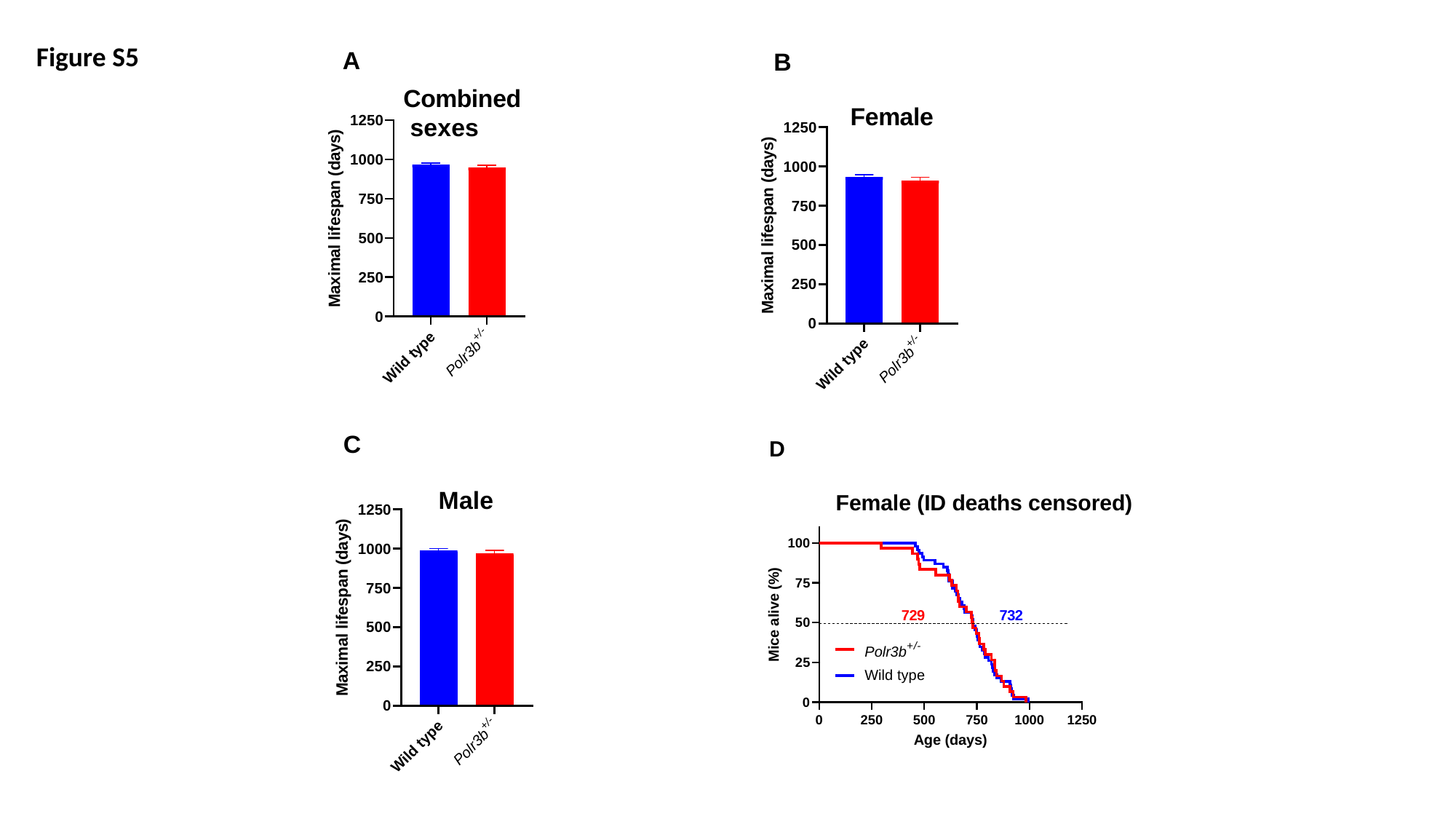

Figure S5
