# Supplementary material for: Polr3b heterozygosity in mice induces both beneficial and deleterious effects on health during ageing with no effect on lifespan
Source: Aging Cell. 2024 Mar 11;23(5):e14141. doi: 10.1111/acel.14141 (PMC11113255; doi:10.1111/acel.14141)
Supplement: Supplementary file 3 — Table S1. Table S2. [file ACEL-23-e14141-s001.pptx]

## Slide 1
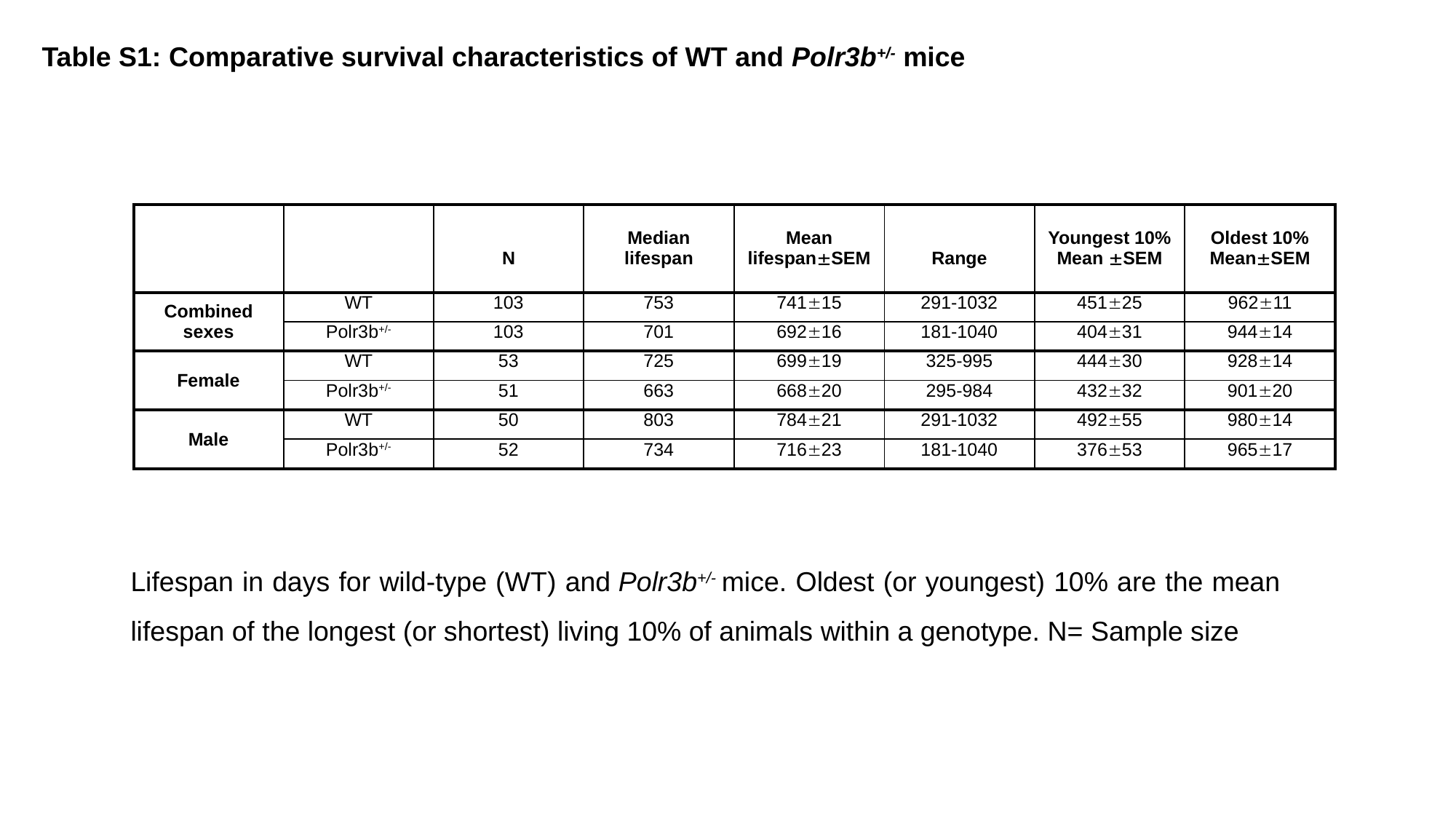

Table S1: Comparative survival characteristics of WT and Polr3b+/- mice
| | | N | Median lifespan | Mean lifespan±SEM | Range | Youngest 10% Mean ±SEM | Oldest 10% Mean±SEM |
| --- | --- | --- | --- | --- | --- | --- | --- |
| Combined sexes | WT | 103 | 753 | 741±15 | 291-1032 | 451±25 | 962±11 |
| | Polr3b+/- | 103 | 701 | 692±16 | 181-1040 | 404±31 | 944±14 |
| Female | WT | 53 | 725 | 699±19 | 325-995 | 444±30 | 928±14 |
| | Polr3b+/- | 51 | 663 | 668±20 | 295-984 | 432±32 | 901±20 |
| Male | WT | 50 | 803 | 784±21 | 291-1032 | 492±55 | 980±14 |
| | Polr3b+/- | 52 | 734 | 716±23 | 181-1040 | 376±53 | 965±17 |
Lifespan in days for wild-type (WT) and Polr3b+/- mice. Oldest (or youngest) 10% are the mean lifespan of the longest (or shortest) living 10% of animals within a genotype. N= Sample size

## Slide 2
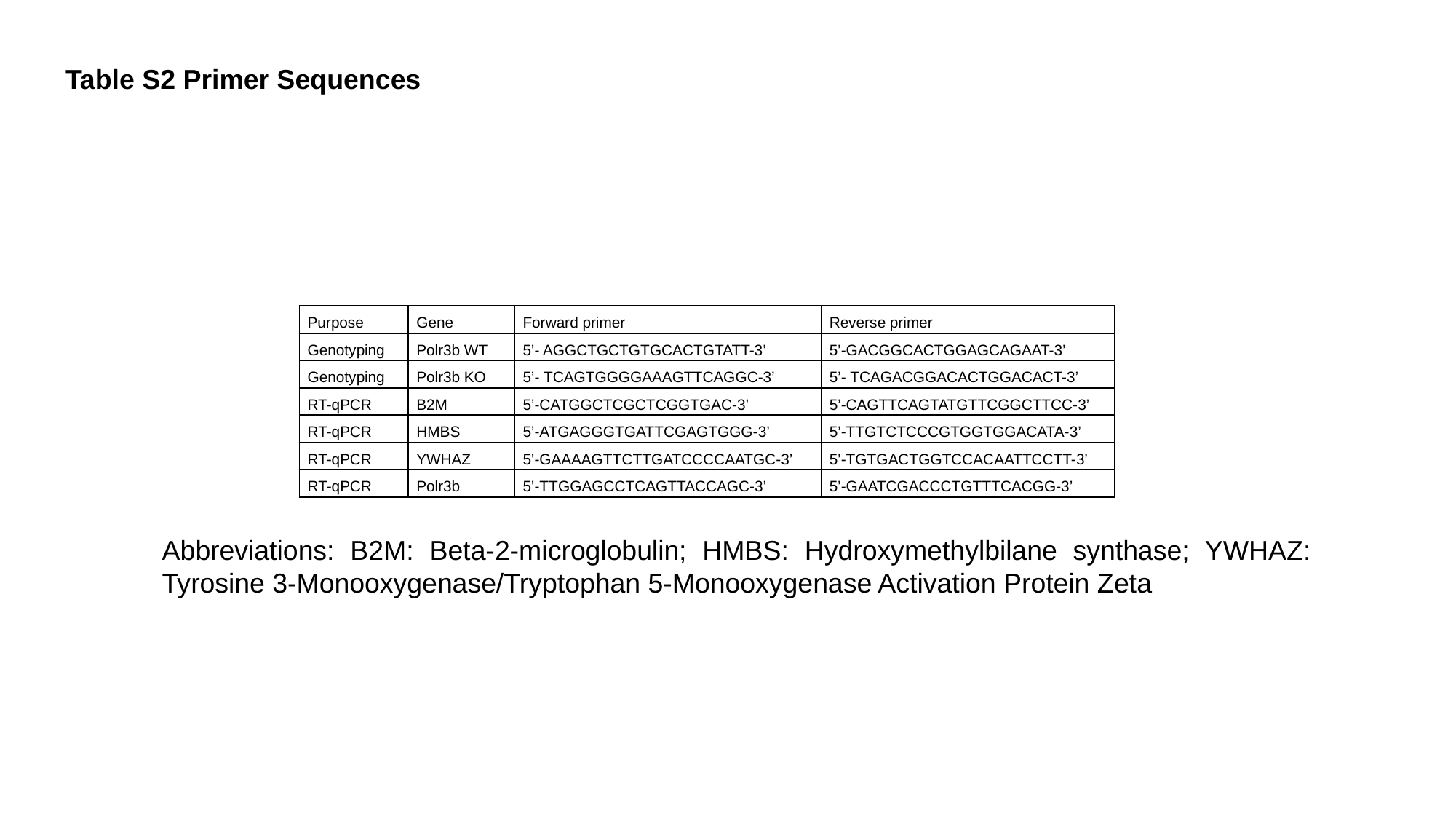

Table S2 Primer Sequences
| Purpose | Gene | Forward primer | Reverse primer |
| --- | --- | --- | --- |
| Genotyping | Polr3b WT | 5’- AGGCTGCTGTGCACTGTATT-3’ | 5’-GACGGCACTGGAGCAGAAT-3’ |
| Genotyping | Polr3b KO | 5’- TCAGTGGGGAAAGTTCAGGC-3’ | 5’- TCAGACGGACACTGGACACT-3’ |
| RT-qPCR | B2M | 5’-CATGGCTCGCTCGGTGAC-3’ | 5’-CAGTTCAGTATGTTCGGCTTCC-3’ |
| RT-qPCR | HMBS | 5’-ATGAGGGTGATTCGAGTGGG-3’ | 5’-TTGTCTCCCGTGGTGGACATA-3’ |
| RT-qPCR | YWHAZ | 5’-GAAAAGTTCTTGATCCCCAATGC-3’ | 5’-TGTGACTGGTCCACAATTCCTT-3’ |
| RT-qPCR | Polr3b | 5’-TTGGAGCCTCAGTTACCAGC-3’ | 5’-GAATCGACCCTGTTTCACGG-3’ |
Abbreviations: B2M: Beta-2-microglobulin; HMBS: Hydroxymethylbilane synthase; YWHAZ: Tyrosine 3-Monooxygenase/Tryptophan 5-Monooxygenase Activation Protein Zeta
