# Supplementary material for: Polr3b heterozygosity in mice induces both beneficial and deleterious effects on health during ageing with no effect on lifespan
Source: Aging Cell. 2024 Mar 11;23(5):e14141. doi: 10.1111/acel.14141 (PMC11113255; doi:10.1111/acel.14141)
Supplement: Supplementary file 4 — Table S3. [file ACEL-23-e14141-s003.pdf]

| Name | Sex | Genotype | Date of Birth | Date of Death | Lifespan (d) |
|---|---|---|---|---|---|
| Polr3b-315 | F | WT | 02/02/2020 | 10/09/2021 | 586 |
| Polr3b-316 | F | WT | 02/02/2020 | 08/09/2021 | 584 |
| Polr3b-317 | F | WT | 02/02/2020 | 03/09/2021 | 579 |
| Polr3b-318 | F | WT | 02/02/2020 | 31/01/2022 | 729 |
| Polr3b-319 | F | WT | 02/02/2020 | 27/01/2022 | 725 |
| Polr3b-320 | F | WT | 02/02/2020 | 03/02/2022 | 732 |
| Polr3b-321 | F | WT | 02/02/2020 | 25/11/2021 | 662 |
| Polr3b-327 | F | WT | 02/02/2020 | 05/08/2022 | 915 |
| Polr3b-328 | F | WT | 02/02/2020 | 14/06/2021 | 498 |
| Polr3b-329 | F | WT | 02/02/2020 | 07/08/2021 | 552 |
| Polr3b-330 | F | WT | 02/02/2020 | 07/03/2022 | 764 |
| Polr3b-341 | F | WT | 02/02/2020 | 13/12/2021 | 680 |
| Polr3b-391 | F | WT | 03/02/2020 | 15/08/2022 | 924 |
| Polr3b-392 | F | WT | 03/02/2020 | 04/03/2022 | 760 |
| Polr3b-393 | F | WT | 03/02/2020 | 12/11/2021 | 648 |
| Polr3b-394 | F | WT | 03/02/2020 | 03/05/2022 | 820 |
| Polr3b-356 | F | WT | 03/02/2020 | 12/10/2021 | 617 |
| Polr3b-357 | F | WT | 03/02/2020 | 11/10/2021 | 616 |
| Polr3b-358 | F | WT | 03/02/2020 | 18/03/2022 | 774 |
| Polr3b-359 | F | WT | 03/02/2020 | 29/10/2021 | 634 |
| Polr3b-360 | F | WT | 03/02/2020 | 21/02/2022 | 749 |
| Polr3b-383 | F | WT | 03/02/2020 | 17/05/2021 | 469 |
| Polr3b-384 | F | WT | 03/02/2020 | 17/02/2022 | 745 |
| Polr3b-385 | F | WT | 03/02/2020 | 08/06/2021 | 491 |
| Polr3b-386 | F | WT | 03/02/2020 | 09/08/2022 | 918 |
| Polr3b-387 | F | WT | 03/02/2020 | 24/05/2021 | 476 |
| Polr3b-778 | F | WT | 10/02/2020 | 31/12/2020 | 325 |
| Polr3b-779 | F | WT | 10/02/2020 | 31/12/2021 | 690 |
| Polr3b-788 | F | WT | 10/02/2020 | 14/05/2021 | 459 |
| Polr3b-789 | F | WT | 10/02/2020 | 13/05/2022 | 823 |
| Polr3b-790 | F | WT | 10/02/2020 | 21/02/2022 | 742 |
| Polr3b-791 | F | WT | 10/02/2020 | 17/05/2022 | 827 |
| Polr3b-792 | F | WT | 10/02/2020 | 05/11/2021 | 634 |
| Polr3b-799 | F | WT | 11/02/2020 | 08/03/2022 | 756 |
| Polr3b-800 | F | WT | 11/02/2020 | 24/06/2022 | 864 |
| Polr3b-801 | F | WT | 11/02/2020 | 25/05/2022 | 834 |
| Polr3b-802 | F | WT | 11/02/2020 | 06/06/2022 | 846 |
| Polr3b-803 | F | WT | 11/02/2020 | 02/03/2022 | 750 |
| Polr3b-804 | F | WT | 11/02/2020 | 02/11/2022 | 995 |
| Polr3b-805 | F | WT | 11/02/2020 | 27/04/2022 | 806 |
| Polr3b-814 | F | WT | 16/02/2020 | 07/10/2021 | 599 |
| Polr3b-815 | F | WT | 16/02/2020 | 29/09/2021 | 591 |
| Polr3b-816 | F | WT | 16/02/2020 | 15/04/2022 | 789 |
| Polr3b-817 | F | WT | 16/02/2020 | 05/07/2021 | 505 |
| Polr3b-822 | F | WT | 16/02/2020 | 15/08/2022 | 911 |
| Polr3b-823 | F | WT | 16/02/2020 | 17/02/2022 | 732 |
| Polr3b-824 | F | WT | 16/02/2020 | 16/12/2021 | 669 |
| Polr3b-825 | F | WT | 16/02/2020 | 03/12/2021 | 656 |
| Polr3b-826 | F | WT | 16/02/2020 | 07/01/2022 | 691 |
| Polr3b-827 | F | WT | 16/02/2020 | 22/10/2021 | 614 |
| Polr3b-828 | F | WT | 16/02/2020 | 11/04/2022 | 785 |
| Polr3b-829 | F | WT | 16/02/2020 | 18/10/2021 | 610 |
| Polr3b-830 | F | WT | 16/02/2020 | 11/08/2022 | 907 |
| | | | | | |
| Polr3b-170 | F | Het | 02/02/2020 | 21/01/2022 | 719 |
| Polr3b-172 | F | Het | 02/02/2020 | 26/11/2021 | 663 |
| Polr3b-189 | F | Het | 02/02/2020 | 23/11/2020 | 295 |
| Polr3b-190 | F | Het | 02/02/2020 | 01/04/2022 | 789 |

| | | | | | |
|---|---|---|---|---|---|
| Polr3b-192 | F | Het | 02/02/2020 | 15/10/2021 | 621 |
| Polr3b-199 | F | Het | 03/02/2020 | 08/12/2021 | 674 |
| Polr3b-202 | F | Het | 03/02/2020 | 20/06/2022 | 868 |
| Polr3b-216 | F | Het | 03/02/2020 | 20/05/2022 | 837 |
| Polr3b-217 | F | Het | 03/02/2020 | 28/01/2022 | 725 |
| Polr3b-218 | F | Het | 03/02/2020 | 12/11/2021 | 648 |
| Polr3b-234 | F | Het | 03/02/2020 | 07/03/2022 | 763 |
| Polr3b-236 | F | Het | 03/02/2020 | 19/05/2022 | 836 |
| Polr3b-237 | F | Het | 03/02/2020 | 04/03/2022 | 760 |
| Polr3b-243 | F | Het | 03/02/2020 | 30/06/2022 | 878 |
| Polr3b-244 | F | Het | 03/02/2020 | 26/10/2021 | 631 |
| Polr3b-259 | F | Het | 04/02/2020 | 14/06/2021 | 496 |
| Polr3b-260 | F | Het | 04/02/2020 | 14/07/2021 | 526 |
| Polr3b-263 | F | Het | 04/02/2020 | 04/05/2022 | 820 |
| Polr3b-266 | F | Het | 04/02/2020 | 16/11/2021 | 651 |
| Polr3b-268 | F | Het | 04/02/2020 | 31/01/2022 | 727 |
| Polr3b-280 | F | Het | 04/02/2020 | 05/01/2022 | 701 |
| Polr3b-284 | F | Het | 04/02/2020 | 02/06/2021 | 484 |
| Polr3b-285 | F | Het | 04/02/2020 | 27/05/2021 | 478 |
| Polr3b-293 | F | Het | 04/02/2020 | 26/11/2021 | 661 |
| Polr3b-302 | F | Het | 06/02/2020 | 05/07/2021 | 515 |
| Polr3b-304 | F | Het | 06/02/2020 | 19/05/2021 | 468 |
| Polr3b-425 | F | Het | 07/02/2020 | 15/06/2021 | 494 |
| Polr3b-428 | F | Het | 07/02/2020 | 25/05/2021 | 473 |
| Polr3b-434 | F | Het | 07/02/2020 | 31/05/2022 | 844 |
| Polr3b-436 | F | Het | 07/02/2020 | 22/02/2022 | 746 |
| Polr3b-457 | F | Het | 09/02/2020 | 04/08/2022 | 907 |
| Polr3b-460 | F | Het | 09/02/2020 | 04/03/2022 | 754 |
| Polr3b-485 | F | Het | 09/02/2020 | 29/09/2021 | 598 |
| Polr3b-486 | F | Het | 09/02/2020 | 23/07/2021 | 530 |
| Polr3b-473 | F | Het | 10/02/2020 | 10/02/2022 | 731 |
| Polr3b-474 | F | Het | 10/02/2020 | 21/10/2022 | 984 |
| Polr3b-475 | F | Het | 10/02/2020 | 19/08/2021 | 556 |
| Polr3b-478 | F | Het | 10/02/2020 | 18/08/2021 | 555 |
| Polr3b-493 | F | Het | 12/02/2020 | 22/04/2022 | 800 |
| Polr3b-516 | F | Het | 14/02/2020 | 26/11/2021 | 651 |
| Polr3b-517 | F | Het | 14/02/2020 | 06/04/2022 | 782 |
| Polr3b-518 | F | Het | 14/02/2020 | 29/10/2021 | 623 |
| Polr3b-519 | F | Het | 14/02/2020 | 19/11/2021 | 644 |
| Polr3b-520 | F | Het | 14/02/2020 | 14/06/2021 | 486 |
| Polr3b-526 | F | Het | 15/02/2020 | 08/09/2021 | 571 |
| Polr3b-527 | F | Het | 15/02/2020 | 05/05/2021 | 445 |
| Polr3b-542 | F | Het | 15/02/2020 | 13/12/2021 | 667 |
| Polr3b-543 | F | Het | 15/02/2020 | 26/08/2022 | 923 |
| Polr3b-556 | F | Het | 15/02/2020 | 07/02/2022 | 723 |
| Polr3b-558 | F | Het | 15/02/2020 | 29/11/2021 | 653 |
| Polr3b-551 | F | Het | 16/02/2020 | 19/01/2022 | 703 |
| | | | | | |
| Polr3b-307 | M | WT | 02/02/2020 | 19/01/2022 | 717 |
| Polr3b-308 | M | WT | 02/02/2020 | 09/03/2022 | 766 |
| Polr3b-309 | M | WT | 02/02/2020 | 27/08/2021 | 572 |
| Polr3b-312 | M | WT | 02/02/2020 | 28/03/2022 | 785 |
| Polr3b-313 | M | WT | 02/02/2020 | 09/05/2022 | 827 |
| Polr3b-314 | M | WT | 02/02/2020 | 24/02/2022 | 753 |
| Polr3b-322 | M | WT | 02/02/2020 | 19/11/2020 | 291 |
| Polr3b-323 | M | WT | 02/02/2020 | 17/05/2022 | 835 |
| Polr3b-324 | M | WT | 02/02/2020 | 22/07/2022 | 901 |
| Polr3b-325 | M | WT | 02/02/2020 | 01/06/2022 | 850 |
| Polr3b-326 | M | WT | 02/02/2020 | 01/08/2022 | 911 |

| | | | | | |
|---|---|---|---|---|---|
| Polr3b-339 | M | WT | 02/02/2020 | 30/05/2022 | 848 |
| Polr3b-340 | M | WT | 03/02/2020 | 06/12/2021 | 672 |
| Polr3b-351 | M | WT | 03/02/2020 | 01/03/2022 | 757 |
| Polr3b-352 | M | WT | 03/02/2020 | 18/08/2022 | 927 |
| Polr3b-354 | M | WT | 03/02/2020 | 12/08/2022 | 921 |
| Polr3b-355 | M | WT | 03/02/2020 | 26/09/2022 | 966 |
| Polr3b-374 | M | WT | 03/02/2020 | 21/04/2022 | 808 |
| Polr3b-375 | M | WT | 03/02/2020 | 26/09/2022 | 966 |
| Polr3b-376 | M | WT | 03/02/2020 | 22/04/2022 | 809 |
| Polr3b-378 | M | WT | 03/02/2020 | 22/08/2022 | 931 |
| Polr3b-379 | M | WT | 03/02/2020 | 08/04/2022 | 795 |
| Polr3b-381 | M | WT | 03/02/2020 | 22/06/2022 | 870 |
| Polr3b-382 | M | WT | 03/02/2020 | 01/12/2022 | 1032 |
| Polr3b-767 | M | WT | 10/02/2020 | 16/07/2021 | 522 |
| Polr3b-768 | M | WT | 10/02/2020 | 12/04/2022 | 792 |
| Polr3b-769 | M | WT | 10/02/2020 | 10/11/2022 | 1004 |
| Polr3b-770 | M | WT | 10/02/2020 | 12/11/2021 | 641 |
| Polr3b-771 | M | WT | 10/02/2020 | 30/12/2021 | 689 |
| Polr3b-772 | M | WT | 10/02/2020 | 18/04/2022 | 798 |
| Polr3b-773 | M | WT | 10/02/2020 | 28/02/2022 | 749 |
| Polr3b-774 | M | WT | 10/02/2020 | 25/07/2022 | 896 |
| Polr3b-775 | M | WT | 10/02/2020 | 10/03/2022 | 759 |
| Polr3b-776 | M | WT | 10/02/2020 | 02/09/2022 | 935 |
| Polr3b-777 | M | WT | 10/02/2020 | 15/11/2021 | 644 |
| Polr3b-780 | M | WT | 10/02/2020 | 29/04/2022 | 809 |
| Polr3b-781 | M | WT | 10/02/2020 | 01/11/2021 | 630 |
| Polr3b-782 | M | WT | 10/02/2020 | 04/05/2022 | 814 |
| Polr3b-783 | M | WT | 10/02/2020 | 22/12/2021 | 681 |
| Polr3b-784 | M | WT | 10/02/2020 | 18/08/2022 | 920 |
| Polr3b-785 | M | WT | 10/02/2020 | 15/11/2021 | 644 |
| Polr3b-786 | M | WT | 10/02/2020 | 01/10/2021 | 599 |
| Polr3b-787 | M | WT | 10/02/2020 | 21/01/2022 | 711 |
| Polr3b-793 | M | WT | 11/02/2020 | 31/05/2021 | 475 |
| Polr3b-794 | M | WT | 11/02/2020 | 02/08/2022 | 903 |
| Polr3b-795 | M | WT | 11/02/2020 | 01/06/2022 | 841 |
| Polr3b-796 | M | WT | 11/02/2020 | 30/08/2022 | 931 |
| Polr3b-797 | M | WT | 11/02/2020 | 29/10/2021 | 626 |
| Polr3b-798 | M | WT | 11/02/2020 | 13/10/2022 | 975 |
| Polr3b-806 | M | WT | 16/02/2020 | 08/02/2022 | 723 |
| | | | | | |
| Polr3b-177 | M | Het | 02/02/2020 | 05/10/2021 | 611 |
| Polr3b-178 | M | Het | 02/02/2020 | 10/05/2021 | 463 |
| Polr3b-181 | M | Het | 02/02/2020 | 19/04/2022 | 807 |
| Polr3b-184 | M | Het | 02/02/2020 | 28/12/2021 | 695 |
| Polr3b-186 | M | Het | 02/02/2020 | 30/08/2022 | 940 |
| Polr3b-188 | M | Het | 02/02/2020 | 30/12/2021 | 697 |
| Polr3b-195 | M | Het | 03/02/2020 | 01/09/2022 | 941 |
| Polr3b-198 | M | Het | 03/02/2020 | 01/06/2022 | 849 |
| Polr3b-204 | M | Het | 03/02/2020 | 24/02/2022 | 752 |
| Polr3b-205 | M | Het | 03/02/2020 | 21/04/2022 | 808 |
| Polr3b-210 | M | Het | 03/02/2020 | 12/11/2021 | 648 |
| Polr3b-212 | M | Het | 03/02/2020 | 01/11/2021 | 637 |
| Polr3b-221 | M | Het | 03/02/2020 | 13/10/2022 | 983 |
| Polr3b-223 | M | Het | 03/02/2020 | 25/02/2021 | 388 |
| Polr3b-225 | M | Het | 03/02/2020 | 27/07/2022 | 905 |
| Polr3b-228 | M | Het | 03/02/2020 | 11/03/2022 | 767 |
| Polr3b-229 | M | Het | 03/02/2020 | 08/03/2022 | 764 |
| Polr3b-231 | M | Het | 03/02/2020 | 25/05/2021 | 477 |
| Polr3b-233 | M | Het | 03/02/2020 | 18/03/2022 | 774 |

| | | | | | |
|---|---|---|---|---|---|
| Polr3b-239 | M | Het | 03/02/2020 | 25/08/2022 | 934 |
| Polr3b-242 | M | Het | 03/02/2020 | 19/01/2022 | 716 |
| Polr3b-251 | M | Het | 04/02/2020 | 11/06/2021 | 493 |
| Polr3b-252 | M | Het | 04/02/2020 | 24/11/2021 | 659 |
| Polr3b-253 | M | Het | 04/02/2020 | 23/02/2022 | 750 |
| Polr3b-255 | M | Het | 04/02/2020 | 14/02/2022 | 741 |
| Polr3b-258 | M | Het | 04/02/2020 | 13/06/2022 | 860 |
| Polr3b-269 | M | Het | 04/02/2020 | 15/07/2021 | 527 |
| Polr3b-273 | M | Het | 04/02/2020 | 03/08/2020 | 181 |
| Polr3b-274 | M | Het | 04/02/2020 | 03/12/2021 | 668 |
| Polr3b-278 | M | Het | 04/02/2020 | 25/06/2021 | 507 |
| Polr3b-429 | M | Het | 07/02/2020 | 02/05/2022 | 815 |
| Polr3b-433 | M | Het | 07/02/2020 | 25/11/2021 | 657 |
| Polr3b-441 | M | Het | 09/02/2020 | 30/12/2021 | 690 |
| Polr3b-442 | M | Het | 09/02/2020 | 17/02/2022 | 739 |
| Polr3b-443 | M | Het | 09/02/2020 | 02/05/2022 | 813 |
| Polr3b-448 | M | Het | 09/02/2020 | 22/12/2021 | 682 |
| Polr3b-449 | M | Het | 09/02/2020 | 20/06/2022 | 862 |
| Polr3b-450 | M | Het | 09/02/2020 | 15/12/2022 | 1040 |
| Polr3b-479 | M | Het | 09/02/2020 | 15/09/2022 | 949 |
| Polr3b-480 | M | Het | 09/02/2020 | 17/12/2021 | 677 |
| Polr3b-482 | M | Het | 09/02/2020 | 16/02/2021 | 373 |
| Polr3b-437 | M | Het | 10/02/2020 | 01/06/2022 | 842 |
| Polr3b-439 | M | Het | 10/02/2020 | 20/06/2022 | 861 |
| Polr3b-440 | M | Het | 10/02/2020 | 26/07/2021 | 532 |
| Polr3b-467 | M | Het | 10/02/2020 | 17/03/2022 | 766 |
| Polr3b-468 | M | Het | 10/02/2020 | 01/06/2022 | 842 |
| Polr3b-469 | M | Het | 10/02/2020 | 04/02/2022 | 725 |
| Polr3b-470 | M | Het | 10/02/2020 | 07/02/2022 | 728 |
| Polr3b-471 | M | Het | 10/02/2020 | 30/09/2021 | 598 |
| Polr3b-487 | M | Het | 12/02/2020 | 01/10/2021 | 597 |
| Polr3b-488 | M | Het | 12/02/2020 | 31/05/2022 | 839 |
| Polr3b-496 | M | Het | 12/02/2020 | 15/11/2021 | 642 |
